# Spaceflight Induces Strength Decline in *Caenorhabditis elegans*

**DOI:** 10.3390/cells12202470

**Published:** 2023-10-17

**Authors:** Purushottam Soni, Hunter Edwards, Taslim Anupom, Mizanur Rahman, Leila Lesanpezeshki, Jerzy Blawzdziewicz, Henry Cope, Nima Gharahdaghi, Daniel Scott, Li Shean Toh, Philip M. Williams, Timothy Etheridge, Nathaniel Szewczyk, Craig R. G. Willis, Siva A. Vanapalli

**Affiliations:** 1 Department of Chemical Engineering, Texas Tech University, Lubbock, TX 79409, USA; soni@rose-hulman.edu (P.S.); mizanur.rahman@nemalifeinc.com (M.R.); pezeshki.lesan@gmail.com (L.L.); 2 Department of Biological Sciences, Texas Tech University, Lubbock, TX 79409, USA; hunter.edwards@ttu.edu; 3 Department of Electrical Engineering, Texas Tech University, Lubbock, TX 79409, USA; taslim.anupom@nemalifeinc.com; 4 Department of Mechanical Engineering, Texas Tech University, Lubbock, TX 79409, USA; jerzy.blawzdziewicz@ttu.edu; 5 Department of Physics and Astronomy, Texas Tech University, Lubbock, TX 79409, USA; 6 School of Medicine, University of Nottingham, Derby DE22 3DT, UK; henry.cope@nottingham.ac.uk (H.C.); nima.gharahdaghi@ndm.ox.ac.uk (N.G.); 7 School of Life Sciences, University of Nottingham, Nottingham NG7 2UH, UK; daniel.scott@nottingham.ac.uk; 8 School of Pharmacy, University of Nottingham, Nottingham NG7 2RD, UK; lishean.toh@nottingham.ac.uk (L.S.T.); phil.williams@nottingham.ac.uk (P.M.W.); 9 Department of Sport and Health Sciences, College of Life and Environmental Sciences, University of Exeter, Exeter EX1 2LU, UK; t.etheridge@exeter.ac.uk; 10 Ohio Musculoskeletal and Neurological Institute, Heritage College of Osteopathic Medicine, Ohio University, Athens, OH 45701, USA; 11 School of Chemistry and Biosciences, Faculty of Life Sciences, University of Bradford, Bradford BD7 1DP, UK; c.r.g.willis@bradford.ac.uk

**Keywords:** *C. elegans*, microgravity, muscle strength, muscle atrophy, spaceflight, dystrophin, International Space Station, omics, gene expression, astropharmacy

## Abstract

**Background:** Understanding and countering the well-established negative health consequences of spaceflight remains a primary challenge preventing safe deep space exploration. Targeted/personalized therapeutics are at the forefront of space medicine strategies, and cross-species molecular signatures now define the ‘typical’ spaceflight response. However, a lack of direct genotype–phenotype associations currently limits the robustness and, therefore, the therapeutic utility of putative mechanisms underpinning pathological changes in flight. **Methods:** We employed the worm *Caenorhabditis elegans* as a validated model of space biology, combined with ‘NemaFlex-S’ microfluidic devices for assessing animal strength production as one of the most reproducible physiological responses to spaceflight. Wild-type and *dys-1* (BZ33) strains (a Duchenne muscular dystrophy (DMD) model for comparing predisposed muscle weak animals) were cultured on the International Space Station in chemically defined media before loading second-generation gravid adults into NemaFlex-S devices to assess individual animal strength. These same cultures were then frozen on orbit before returning to Earth for next-generation sequencing transcriptomic analysis. **Results:** Neuromuscular strength was lower in flight *versus* ground controls (16.6% decline, *p* < 0.05), with *dys-1* significantly more (23% less strength, *p* < 0.01) affected than wild types. The transcriptional gene ontology signatures characterizing both strains of weaker animals in flight strongly corroborate previous results across species, enriched for upregulated stress response pathways and downregulated mitochondrial and cytoskeletal processes. Functional gene cluster analysis extended this to implicate decreased neuronal function, including abnormal calcium handling and acetylcholine signaling, in space-induced strength declines under the predicted control of UNC-89 and DAF-19 transcription factors. Finally, gene modules specifically altered in *dys-1* animals in flight again cluster to neuronal/neuromuscular pathways, suggesting strength loss in DMD comprises a strong neuronal component that predisposes these animals to exacerbated strength loss in space. **Conclusions:** Highly reproducible gene signatures are strongly associated with space-induced neuromuscular strength loss across species and neuronal changes in calcium/acetylcholine signaling require further study. These results promote targeted medical efforts towards and provide an in vivo model for safely sending animals and people into deep space in the near future.

## 1. Introduction

As a species, we have been working and living in space for more than 60 years, with continuous occupation for the last 23 years via the International Space Station (ISS) [1]. During this time, we have discovered that spaceflight induces numerous physiologic alterations, including but not limited to muscle and bone loss, structural changes in the eyes and brain, altered cardiovascular function, and altered cognitive function [2,3,4,5,6,7,8,9]. In some cases, we have developed effective countermeasures for these undesirable outcomes, but in other cases, understanding and countering the causes of these outcomes remain active areas of investigation. For example, adequate exercise and nutrition are sufficient to limit bone and muscle loss in astronauts onboard the ISS [10]. In contrast, causes of and countermeasures for alterations in the brain remain active areas of research [11]. As research advances, it has become increasingly clear that there are some molecular hallmarks of spaceflight [12], which include mitochondrial dysregulation, oxidative stress, DNA damage, and epigenetic changes. Current challenges of space life sciences research include linking these molecular alterations to the physiologic ones as well as to the known spaceflight hazards. These hazards include distance from Earth, confinement, being in a hostile and closed environment, altered gravity, and increased and altered radiation exposure [12,13,14].

With our increased presence in space, our ability to conduct space medicine continues to improve. For example, we now understand that improved skin care is needed in flight due to the frequency of skin rashes [15]. Currently, there is an increased interest in personalized approaches for optimizing astronaut health [16,17,18,19,20]. For example, exercise and nutrition can be individually tailored to prevent muscle loss in flight [10,21]. The success of these approaches on Earth, combined with the increased use of -omics data in terrestrial medicine, has also led to using -omics approaches in astronauts [17,21,22,23]. While still in its infancy [24,25,26], it seems clear that combining -omics data from space-flown model organisms, such as *C. elegans*, *Drosophila*, and rodents, with astronaut data can accelerate the discovery process, in part by compensating for the current rarity of human space -omics datasets [22,27]. For example, *C. elegans*, rodents, and humans all display alterations in insulin-linked gene expression in response to spaceflight [28]. Given the central role of insulin in human health and longevity on Earth [29], it is highly likely this system is equally important to maintain in space. Indeed, recent data from space-flown rodents confirm that the liver–muscle axis is central to regulating both global metabolic health and muscle health functions the same in space as on Earth and that its perturbation may contribute to both liver and muscle pathology in flight [30].

While our advances in fundamental space life sciences and space medicine have been substantial, these are largely limited to habitation in the Low Earth Orbit (LEO). With planned government and commercial missions beyond LEO (BLEO), understanding fundamental changes to biological systems and countermeasures to detrimental alterations at new destinations, such as the Moon and Mars, are emerging challenges [31]. For example, current exercise countermeasures for maintaining muscle on the ISS are not feasible on currently planned spacecraft due to size constraints [32]. Similarly, storing adequate nutrition further away from Earth is operationally challenging [33,34,35]. Thus, despite not being a current priority for research on the ISS, understanding molecular mechanisms underpinning muscle atrophy in space remains important for these new exploration class missions.

Currently, designing space biology experiments for BLEO means that the experiments must be small and autonomous [36,37,38]. For example, using a CubeSat, such as Biosentinel [39], as a passive or mostly passive payload on Artemis [40], or an autonomous instrument on a Commercial Lunar Payload Services mission [41]. Thus, using standard flight genomic model organisms [27] that are small, such as bacteria, yeast, *C. elegans*, or *Drosophila,* is feasible. In terms of muscle strength, a microfluidic device has been developed for assessing strength in *C. elegans* [42], and this has been successfully used to both demonstrate decreased strength in mutants with defective muscle structure [43,44,45] and to identify drugs to improve strength both in muscle mutants [46] and with age [47]. Thus, the use of *C. elegans* for studying and countering muscle strength decline on the Moon is technically feasible, albeit challenging.

The use of *C. elegans* in space biology has recently been reviewed [48,49,50]. Notably, past studies have demonstrated conserved gene expression changes in response to spaceflight between *C. elegans*, rodents, and humans. These include declines in muscle contractile genes [51], mitochondrial genes [12,52], and insulin signaling [28,53]. The knockdown of these genes on Earth is sufficient to induce strength decline [45,46]. Therefore, we hypothesized that worms would be weaker in space and aimed to directly test this on the micro-16 ISS payload, flown starting in February 2021 (NG-15 launch). The results from our experiment pave the way for testing pharmaceutical and nutrient countermeasures for strength decline in spaceflown *C. elegans,* as well as for testing *C. elegans* muscle strength at various BLEO destinations.

## 2. Materials and Methods

### 2.1. Worm Preparation

**Figure 1:**
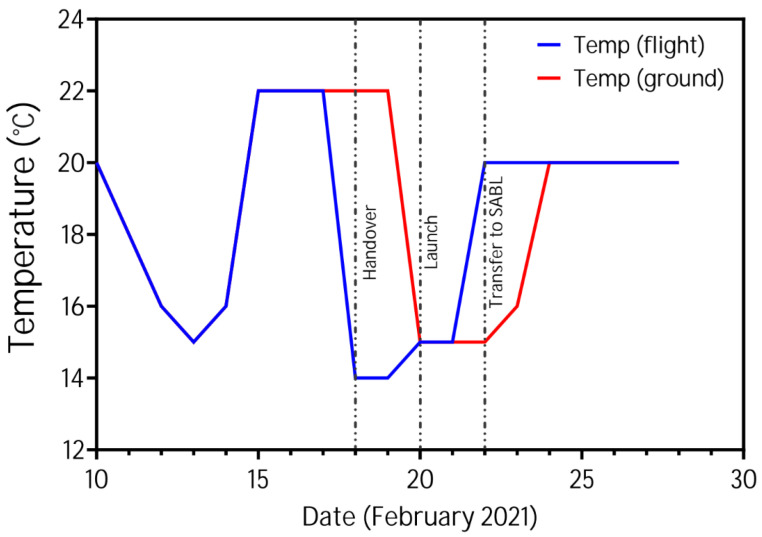
Detailed temperature profile of the culture bags during shipping from TTU to EVMS and Launch. Bags were stored at 20 °C after receiving them at ISS and TTU Lab.

Wild-type (*wt*) Bristol isolate (N2 [54]) and dystrophin (*dys-1*) mutant (BZ33 [55]) worms were maintained in *C. elegans* Maintenance Medium (CeMM) [56], purchased on contract from Cell Guidance Systems, Cambridge, UK. Cultures were established as previously described [49] and maintained at two separate sites (Texas Tech University, USA and University of Nottingham, UK). Ten days before the launch, approximately 1000 larvae from a stock culture were transferred into a Fluorinated Ethylene Propylene (FEP) bag (Saint Gobain Performance Plastics Corporation, Solon, OH, USA) containing 20 mL CeMM at Texas Tech University (TTU). These primary culture bags and CeMM-Filled FEP bags were shipped to the Eastern Virginia Medical School (EVMS), Norfolk, on 10th February 2021. The culture bags were shipped with phase change material [57] at a temperature of 16 ± 2 °C. Upon arrival (following a lengthy delay of shipment in transit due to poor weather), culture bags were inspected for contamination and stored in the incubator at 22 °C for 2 days (flight bags) and 4 days (ground bags). The flight bags were handed over to NASA cold stowage on 17th February 2021 at a temperature of 15 ± 1 °C for the 20th February 2021 NG-15 launch. Ground control bags were sent to TTU, and the temperature profile of flight bags was replicated on a time delay of two days (Figure 1). Upon arrival at the ISS, the culture bags were incubated at 20 °C, and the CeMM bags were stored at 4 °C. Cultures were incubated for a week in microgravity before initiating the multigenerational culturing outlined in [49]. Briefly, 1 mL of culture was transferred to a fresh FEP bag containing CeMM every 2 weeks. All experiments used a starting mixed population of well-fed animals, with force measurements only being made on gravid adults. Upon experiment completion, ground bags at TTU were frozen and stored at −80 °C, while flight bags were frozen in the ISS MELFI at −80 °C until download to Earth via NASA cold stowage at −20 °C, followed by dry ice transport to TTU for storage at −80 °C.

### 2.2. Fabrication and Assembly of a Microfluidic Device in Worm Loading Apparatus (WLA)

Devices were fabricated and assembled in WLA as previously described [42,49]. The micropillar-based NemaFlex-S device was fabricated using a modified two-step soft lithography process [58]. The mold was fabricated in SU-8 2050 negative photoresist (Microchem) on a 4″ silicon wafer as a substrate. First, a 20 μm tall photoresist layer was fabricated, which forms the boundary of the NemaFlex chamber. Next, a second layer of 80 μm height was fabricated on top of the initial layer. The second layer was fabricated with cylindrical holes that form the micropillars. This two-layer approach provided chambers with a depth of approximately 100 μm containing deformable pillars of 80 μm height. Polydimethylsiloxane (PDMS) devices of 4.25 ± 0.25 mm thickness were cast using Sylgard 184 part A (base) and part B (curing agent) 10:1 by weight (Dow Corning) over the SU-8 mold by curing for approximately 2 h at 70 ± 1 °C. Inlet, outlet, and air vent holes were cored with a 1 mm hole puncher (Acuderm, Fort Lauderdale, FL, USA). The devices were thoroughly cleaned with Scotch tape to remove dirt before bonding. The PDMS replica was then treated in an air-plasma cleaner (Harrick Plasma, Ithaca, NY, USA) for 90s and bonded to a 2 × 3 inch glass slide. Bonding was done, ensuring the pillars did not collapse or deform. The bonded devices were immediately placed in an oven for 10 min at 70 ± 1 °C. Devices were then treated with 5 wt% Pluronic F127 (Sigma-Aldrich, St. Louis, MO, USA) for 30 min to prevent any bacterial build-up and reduce bubble formation during worm loading. After incubation, excess Pluronic was removed by washing the devices with DI water. The treated devices were soaked in DI water overnight at 20 ± 1 °C to release any air bubbles from the devices. Fabrication of devices took place at TTU, with devices shipped to EVMS. The microfluidic chips were shipped in Ziploc bags with DI water. Fabrication of WLA took place at BioServe Space Technologies, with WLA shipped to EVMS. Assembly took place at EVMS and was conducted by BioServe Space Technologies (Boulder, CO, USA).

### 2.3. Image Acquisition and Processing

The worms were loaded into NemaFlex-S chambers using a previously described procedure [49]. Briefly, a five-step process involving a syringe pump and NemaFlex-S device priming was used to transfer worms from FEP culture bags into the NemaFlex-S device for imaging in a semi-automated fashion. For on-orbit loading, an additional manual centrifugation of worms toward the loading port of the FEP bags was achieved by having the crew member swing the bag in a circular motion with the injection port pointed away from the crew member. Additionally, loading volumes on orbit were calibrated based upon the results of a training time point prior to the experimental time point and based upon crew member impressions of culture density for the experimental time point vs. the training time point. Once loaded, worms were allowed to habituate to the micropillar arena for approximately 10 min before imaging. One-minute-long videos of crawling worms were acquired with a Nikon inverted microscope (Eclipse TS 100) at 4× magnification with a camera resolution of 1920 × 1080 pixels recorded at 5 frames per second. All videos were recorded at a temperature of 22 ± 2 °C. The recorded movies were processed offline using custom routines written in MATLAB (Mathworks, R2018b) for the quantification of pillar displacements as previously published [42]. Recorded videos were analyzed manually using ImageJ (version 1.48 [59]) for measuring worm diameters at the mid-section and body length at the centerline of the worm. Videos were processed manually for quantification of coiling phenotyping. Adult worms were identified based on their body size and the presence of eggs.

### 2.4. RNA Extraction, Sequencing and Data Pre-Processing

RNA was extracted from frozen worm samples using Direct-zol RNA Miniprep kit (Zymo Research cat# R2050). Two to three independent biological replicates were prepared for each treatment. RNA concentration was determined using Nanodrop 2000 Spectrophotometer (Thermo Fisher Scientific). Library preparation and next-generation sequencing were subsequently performed by the Beijing Genomics Institute (BGI, Hong Kong), with strand-specific (second strand cDNA synthesis with dUTP) 100 bp paired-end reads generated using the DNBseq platform. Cleaned reads (reads with adaptor sequences, contamination, and low-quality reads removed via the SOAPnuke software version 2.1.8 developed by BGI [60]) obtained from BGI were deemed to be of good quality (no over-represented sequences or adapter sequences, and median per base quality scores always >30, as determined using FastQC; Babraham Bioinformatics) and transcript-level abundances consequently estimated via pseudo-alignment to the *C. elegans* reference transcriptome (Ensembl release 108) using Kallisto (version 0.48.0 [61]). Gene counts were then inferred via the tximport R package (version 1.28.0 [62]), and lowly expressed genes were filtered out (genes with a count <10 in every sample) to leave 13,897 genes for downstream analyses.

### 2.5. Gene Expression Analysis

Differential gene expression analysis was performed via DESeq2 (version 1.40.2 [63]) in R (version 4.3.1). Beforehand, principal component analysis (PCA) of the top 500 most variable genes (with variance stabilizing transformed counts used as input) was undertaken for unsupervised clustering of samples. Wald tests were then used to test for differential gene expression, with pairwise comparisons made between flight and ground samples per strain, as well as between the ground samples of each strain and between the flight samples of each strain. Log fold-change shrinkage was performed using an adaptive shrinkage method (ashr) [64], and the Benjamini–Hochberg procedure was used to adjust *p* values to control for false discovery rate (FDR) [65]. Significant gene expression changes in each case were defined at the adjusted *p* < 0.05 level. Functional characteristics of differentially expressed gene lists were elucidated by undertaking over-representation analysis of Gene Ontology (GO) terms using the clusterProfiler R package [66]. In this case, each GO sub-category (biological process, cellular component, molecular function) was considered, with the corresponding background gene list being the genes input into differential expression testing. Enriched GO terms were defined as those with a Benjamini Hochberg corrected *p* < 0.05 [65]. Mapping of our gene expression data to established gene co-expression modules was conducted using the genemodules tool for *C. elegans* [67]. Briefly, this software applied an independent component analysis approach on a large collection of *C. elegans* microarray studies to identify clusters of genes with related gene expression patterns and then annotated these clusters based on their predicted molecular roles and functions. Tool 1 was used per pairwise comparison with log2 fold-change values of all genes subject to differential expression testing (i.e., the 13,897 genes that survived pre-filtering) as input in each case to determine the directional activity of the 209 defined transcriptional modules. Module descriptions potentially relevant to spaceflight and putative transcriptional regulators were subjectively assigned from Supplemental Table S2 (from [67]) and Tool 3. For drug target prediction, differentially expressed genes were first mapped to human orthologs using OrthoList2 [68] with one-to-many mapping. Following this, the QIAGEN Ingenuity Pathway Analysis (IPA) (version 01-22-01) Upstream Regulator Analysis tool [69] was used on the human orthologs of significant differential expressed genes (adjusted *p* < 0.05), with the human orthologs for all genes used as input into differential expression testing serving as the reference/background set. Targets were considered inhibited if the activation Z-score was <−2 and activated if the activation Z-score was >2 [69]. Additionally, targets were only deemed significant if the Benjamini–Hochberg-corrected *p* value was <0.05 [65].

## 3. Results

### 3.1. Growth

Observation of ground control worms indicated that worm growth was as expected based upon results from the experiment verification test [49]. The crew assessed flight growth by eye based on perceived density (e.g., small or medium) of worms within FEP bags. Growth was adequate for the ability to load worms into NemaFlex-S devices as indicated by loading of 37 *wt* including 15 adults and 25 *dys-1*, including 13 adults on the crew’s training time point session. For the experimental time point, 41 *wt* including 30 adults and 49 *dys-1* including 29 adults were loaded into the devices. For comparison, ground cultures for the experimental time point were 45 *wt* including 30 adults and 36 *dys-1* including 25 adults loaded into the devices. These data suggest no major differences in development/reproduction in flight, consistent with past *C. elegans* flight data [70,71,72,73,74,75].

### 3.2. Body Diameter and Length

**Figure 2:**
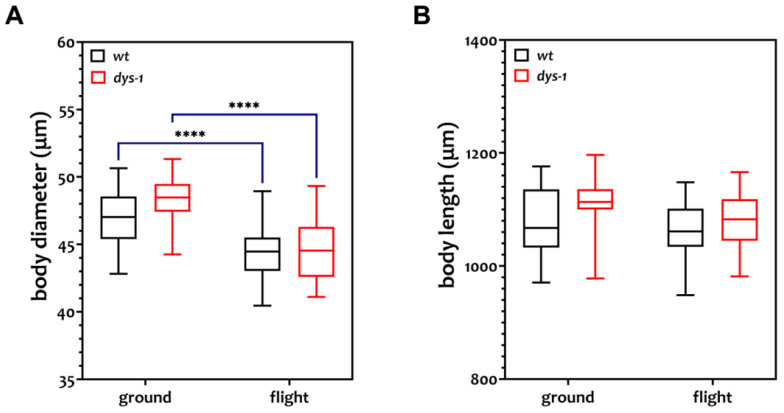
Effect of spaceflight on body diameter and length of *wt* and *dys-1* worms. (**A**) body diameter (**B**) body length. The diameters of both the strains grown at ISS are significantly different compared to ground controls, whereas there is no difference in the length of the worms. Sample size: *n* = 30 for *wt* ground and flight, *n* = 25 for *dys-1* ground, and *n* = 29 for *dys-1* flight. All the data pass the normality test. We used two-way ANOVA (Tukey multiple testing) for calculating significant differences; *p* < 0.001 is for ****.

Force estimation using NemaFlex devices is dependent on adjustment for body diameter [42]. Therefore, the body diameter and length of both *wt* and *dys-1* adult worms were measured from the recorded movies. The diameter of both strains cultured on ISS was significantly lower than both strains cultured on Earth (Figure 2a, Table 1). The body diameter of *wt* worms was 5.5% less, and in *dys-1* worms, it was 7.9% less. The smaller diameter might be due to altered metabolism, as this has previously been reported for spaceflown *C. elegans* [52]. Indeed, our gene expression data are consistent with previously reported gene expression data for spaceflown *C. elegans* [48,51,52,53,76,77]. The length of both strains was not significantly different in flight than on the ground. Note that this contrasts with a previous flight where length decreased significantly by 5.5% [52]. This discrepancy could be because we used mixed populations of worms, whereas [52,78] used age-synchronized worms, or because we are using a different diet, which is known to profoundly impact *C. elegans* morphology and life history [79].

### 3.3. Muscle Strength

**Figure 3:**
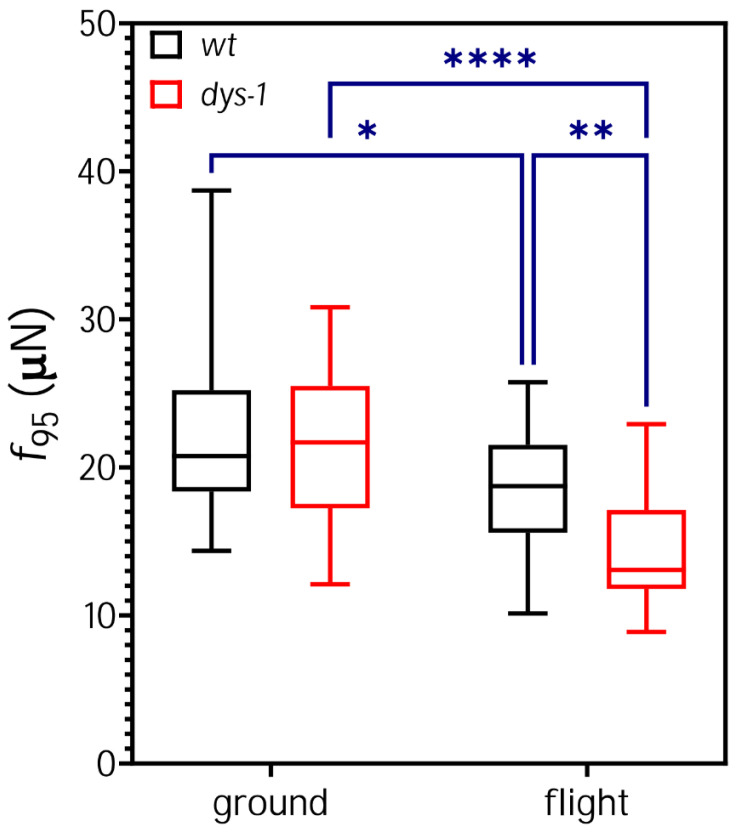
Effect of spaceflight on muscle strength of *wt* and *dys-1* worms. There is no difference in muscle strength between the strains on the ground. The muscle strength of space-grown worms decreased by 16.6% and 33.4% for *wt* and *dys-1*, respectively. Sample size: *n* = 30 for *wt* ground and flight both, *n* = 25 for *dys-1* ground, and *n* = 29 for *dys-1* flight. All the data passed the normality test. We used two-way ANOVA (Tukey multiple testing) for calculating significant differences, * for *p* < 0.05, ** for *p* < 0.01, and **** for *p* < 0.001.

Strength was measured in the second generation in flight to reflect strength in worms wholly developed onboard ISS and with in-flight loading methods and video quality that had been tested on first generation from Earth prior to use with the experimental time point. To measure adult worms’ muscle strength, a 60-s-long video of crawling worms in NemaFlex-S chambers was recorded. The recorded videos were analyzed, and we identified the pillar with the maximal deflection in each image and generated a cumulative probability distribution with all the maximal deflections, as previously described [42]. We use the 95th percentile of this maximal force distribution, referred to as $f_{95}$, as a measure of muscle strength. As shown in Figure 3, on Earth, gravid adult *dys-1* worms are not significantly weaker than *wt*. This is consistent with previous reports where *dys-1* mutants only display strength deficits vs. *wt* post day 1 of adulthood [44].

The muscle strength of the *wt* worms cultured onboard the ISS was significantly lower than on Earth (ground: $f_{95}$ = 22.34 ± 5.67, flight $f_{95}$ = 18.62 ± 4.05, *n* = 30 per group, *p* ≤ 0.05; Figure 3). Notably, the strength deficit (16%) was roughly similar to what is observed in astronauts’ muscles (6–14% [80]) and similar to the previously published quantitative changes in muscle contractile protein during flight (7–10% [51]). In flight, *dys-1* mutants were also weaker than on Earth (ground: $f_{95}$ = 21.38 ± 5.39, *n* = 25; flight $f_{95}$= 14.23 ± 3.87, *n* = 29, *p* ≤ 0.001; Figure 3). It should be noted that the decrease in body diameter of both strains is similar in flight (see Figure 2 and Table 1), indicating the strength decrement is not due to the differences in the measurement technique. The strength in the *dys-1* mutants was 23% less than *wt* worms onboard the ISS. This may reflect our small sample size or a difference in the response to spaceflight in *dys-1* mutants vs. wild-type, as previously reported [81]. It could be that the altered neuromuscular health of *dys-1* worms predisposes them to the negative effects of spaceflight on the neuromuscular system.

### 3.4. Gene Expression Analysis

Past gene expression analysis of spaceflown *C. elegans* has revealed decreased expression of muscle cytoskeletal genes and mitochondrial genes [48,51,52,77,82]. We have previously shown that mutation of some of these muscle genes results in decreased strength [45] and that declines in mitochondrial function also result in decreased strength [46,47], both in *C. elegans* on Earth. Therefore, to confirm that decreased expression of cytoskeletal genes or mitochondrial genes could be contributing to strength decline in flight, we measured gene expression in the same cultures of worms that we measured strength in, as well as two additional cultures.

**Figure 4:**
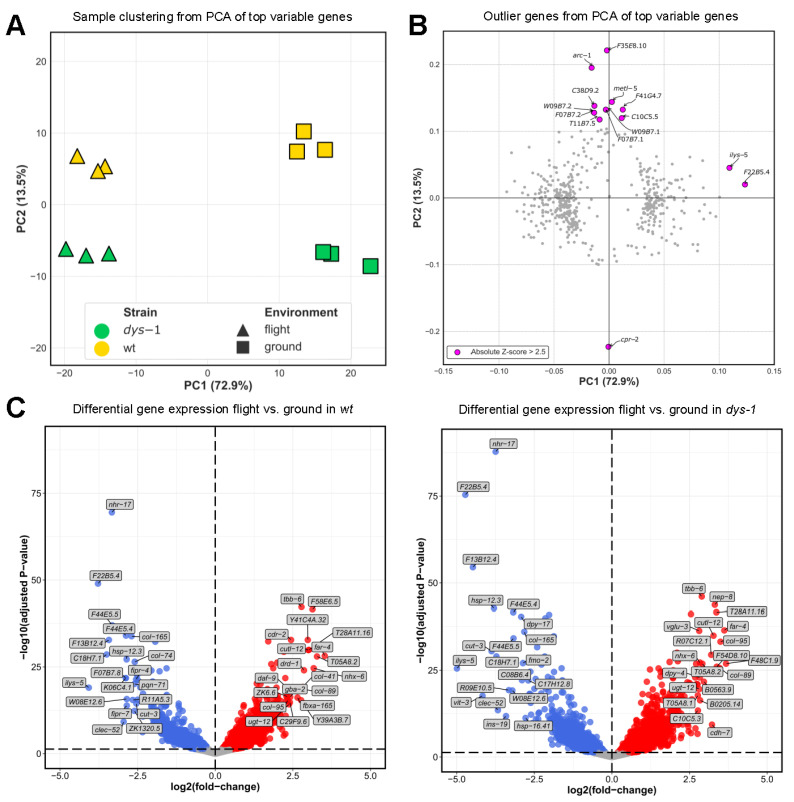
Global trends in spaceflight gene expression. (**A**) PCA clustering of samples based on top 500 most variable genes, (**B**) PCA loadings of top 500 most variable genes, (**C**) Volcano plots for *wt* flight vs. *wt* ground and *dys-1* flight vs. *dys-1* ground differential expression analyses. Annotated genes in each case are those ranked in the top 20 upregulated/downregulated based on log2 fold-change.

Unlike most past flights of *C. elegans* [48], we employed an unbiased approach to the analysis of gene expression (e.g., we did not focus solely on specific subsets of genes). As shown in Figure 4A, Principal Component Analysis (PCA) of the most variable genes showed a distinct clustering of samples in line with their experimental conditions. The tight clustering of samples within conditions indicates that the cultures without strength measures have similar gene expression profiles to the cultures from which strength measures were obtained. The first principal component (PC1), which accounted for 72.9% of the variance, separated the samples based on environment (i.e., flight vs. ground control). Meanwhile, the second principal component (PC2) differentiated the samples based on genetic background, distinguishing between *wt* and *dys-1* mutant and explaining 13.5% of the variance. These results suggest that spaceflight is the main driver of the bulk of gene expression changes, while the *dys-1* mutation has a lesser but significant effect. This is consistent with a past report demonstrating that *dys-1* mutation has an impact on the transcriptional response to spaceflight [81]. The outlier genes driving the variation in the PCA are shown in Figure 4B. The descriptions of these genes match the descriptions of the clusters of genes identified in our module analysis below.

In terms of Differential Expressed Genes (DEGs) with spaceflight, the distribution of expression changes is largely similar between the two strains, as shown in Figure 4C. While it is tempting to examine individual DEGs as meaningful, biological systems are complex, and genes do not act in isolation [83,84]. Therefore, we provide the full set of DEGs (Table S1) but limit discussion of changes to sets of genes with changing expression rather than individual genes.

**Figure 5:**
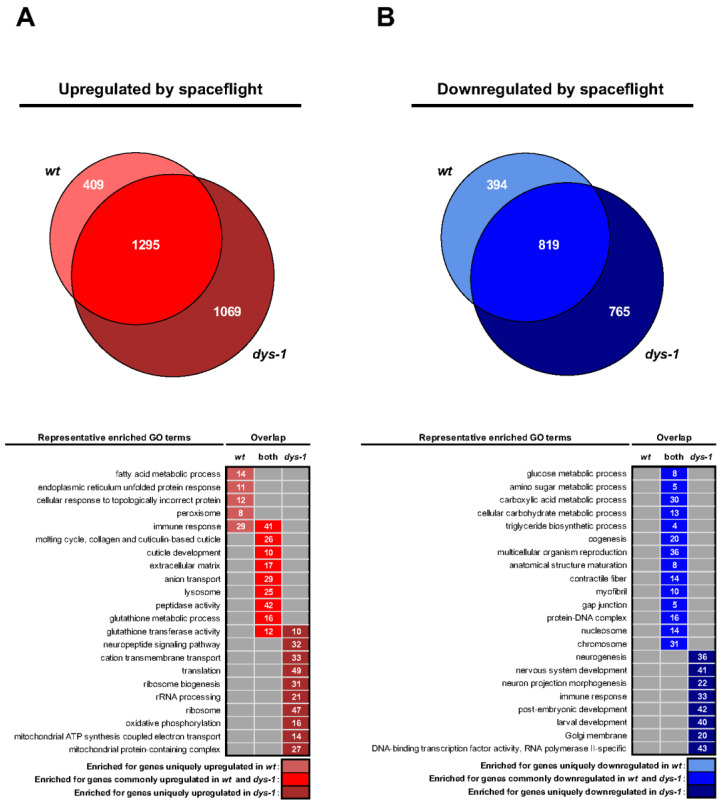
Overlay of *wt* and *dys-1* transcriptome responses to spaceflight. (**A**) Overlay of genes upregulated by spaceflight in *wt* and/or *dys-1* worms. Venn diagram illustrates the degree of commonality/uniqueness in spaceflight-upregulated genes between the two strains, while the heatmap depicts representative Gene Ontology (GO) terms for common/uniquely upregulated genes up in flight. Venn shows the commonality and differential changes in *wt* vs. *dys-1*. The able displays common and differential GO expression in *wt* vs. *dys-1* (**B**) As per panel A but for genes downregulated by spaceflight.

In terms of the genome level scale of changes with spaceflight (e.g., each strain’s flight response normalized against each strain’s ground control), 4726 genes displayed significant changes in expression in response to spaceflight, with 409 uniquely up in *wt*, 394 uniquely down in *wt*, 1069 uniquely up in *dys-1*, 765 uniquely down in *dys-1*, and 1295 up in both strains and 819 down in both strains (Figure 5A).

As shown in Figure 5B, gene ontology analysis of DEGs reveals changes in innate immune response as up in both strains, stress response as up in both strains and metabolism and cytoskeleton as down in both. These changes are broadly similar to past analyses of space-flown *C. elegans* where the stress response is up and metabolic and cytoskeletal genes are down [48,51,52,53,77]. These gene expression changes could underlie the strength decline in flight as strength goes down in both strains. These gene expression changes could also potentially underlie the decrease in muscle size that has previously [85] and recently been reported for other spaceflown *C. elegans* [86]. Changes not likely to underlie strength decline in both strains in flight include upregulation of protein synthesis in *dys-1* in flight and decreased neural development/function in *dys-1* in flight.

**Figure 6:**
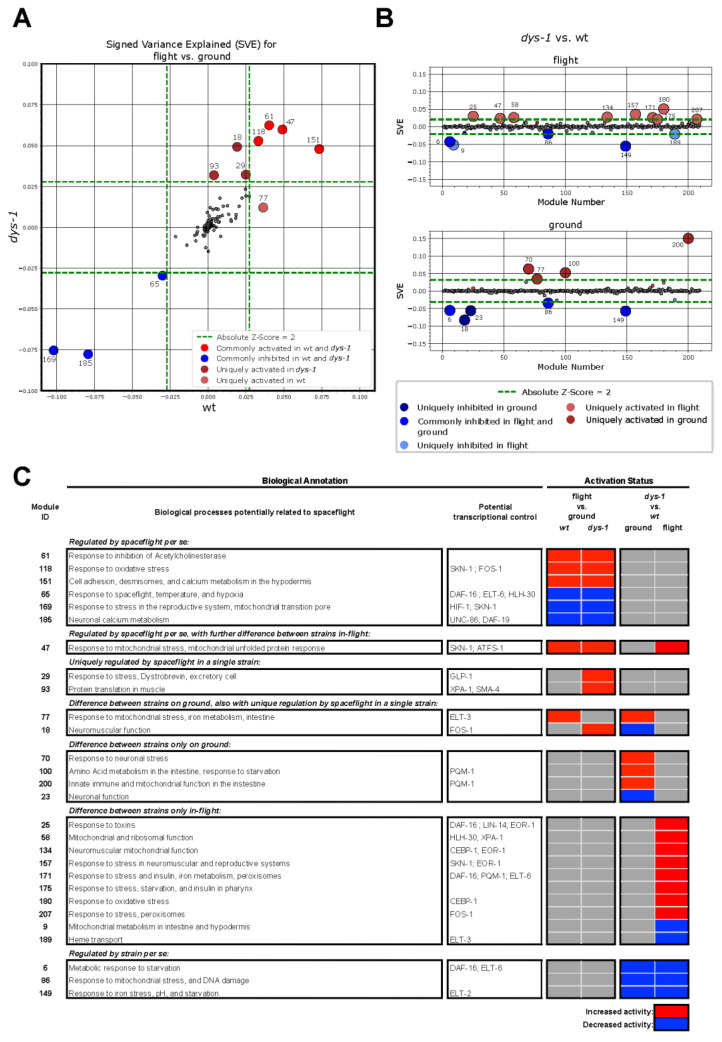
Cluster analysis to identify coexpressed gene modules. (**A**) Activity of gene co-expression modules in flight vs. ground control comparison. (**B**) Activity of gene co-expression modules in *dys-1* vs. *wt* comparisons. (**C**) Table of module annotations and summarized activation between pairwise comparisons.

*C. elegans* was the first multicellular animal for which a transcriptional co-regulation map was generated [87]. This map has recently been updated [67], employing advances in both DEG identification and analysis [88,89,90,91,92]. When *wt* and *dys-1* DEGs are analyzed for the effect of spaceflight vs. ground controls, three modules are inhibited in response to space flight, and four modules are activated in both *wt* and *dys-1* (Figure 6A). Only one module is uniquely activated in *wt,* whereas three are uniquely activated in *dys-1* (Figure 6A). These observations are consistent with spaceflight being the major driver of gene expression changes (Figure 4A). The modules inhibited in both include identifications (Figure 6B) consistent with past spaceflight experiments [48]. Module 65 was previously [67] specifically identified as responsive to spaceflight and other environmental conditions and may be controlled by the transcription factor DAF-16 [93], amongst others, where DAF-16 has been suggested to be a controller of *C. elegans* response to spaceflight [52,53,77,94]. Module 169 is a HIF-1 [95] responsive module [67] and is associated with mitochondrial function. This is consistent with both past *C. elegans* gene expression changes and the recent observation that decreased mitochondrial gene expression is a fundamental feature of biological alterations in response to spaceflight [96]. Notably, this module may also be regulated [67] by SKN-1 [97], which has also previously been suggested to be a transcription factor regulating the response to spaceflight [53]. Module 185 is associated with calcium handling in neurons and may be regulated [67] by UNC-89 [98] and DAF-19 [99]. This module is particularly interesting following recent observations that neuronal morphology [100] and neurotransmitter production [76] are altered in *C. elegans* in flight. Similarly, altered calcium handling in aging *C. elegans* muscle has recently been shown to negatively impact mitochondrial health [101] and, therefore, might be an alternative mechanism by which mitochondrial gene expression declines in response to spaceflight. In terms of commonly activated modules, modules 47 and 118 are both responsive to mitochondrial stress [67], which is consistent with modules 65 and 169 being inhibited. Potential transcriptional regulators of these modules are displayed in (Figure 6C). Module 61 is associated with response to inhibited Acetylcholinesterase [67], which is consistent with Acetylcholinesterase gene expression being decreased in response to spaceflight in *C. elegans* [53]. Module 151 is associated with desmosomal cell adhesion and calcium metabolism in the hypodermis [67]. This result contrasts decreased cell adhesion in *C. elegans* [51,52] and human muscle [102] in response to spaceflight. It could be that this response is unique to *C. elegans* as they have a hydrostatic skeleton that would be predicted to be altered by spaceflight. It could be related to alterations in global calcium homeostasis in *C. elegans* and the potential regulation of mitochondria via both calcium and cadherins in *C. elegans* [47,101], or it could be an over compensation of adhesome structures as a response to failure as recently suggested for aging human muscle [103]. As with the DEG GO analysis, the module analysis identifies decreased expression of mitochondrial metabolism genes and increased expression of mitochondrial/other stress response genes as a potential cause of the decreased strength in flight. Unlike the DEG GO analysis, the module analysis also suggests decreased neuronal function and specifically altered acetylcholine signal from nerve to muscle (and consequent post-synaptic remodeling of excitation–contraction coupling) as another potential contributor to strength decline in flight.

Consistent with the past report that *dys-1* modulates the DEG in response to spaceflight [81], our DEG (Figure 5) and module analysis (Figure 6) confirm *dys-1* modulates the DEG response to spaceflight. As shown in Figure 6A, module 77 is uniquely activated in *wt* and is responsive to mitochondrial stress [67]. Interestingly, module 77 is significantly activated in *dys-1* vs. *wt* on the ground (Figure 6B), potentially explaining the lack of further significant activation in *dys-1* in flight. Activation of this module at baseline is not surprising given the impact of *dys-1* on mitochondrial function [44,46,104] and gene expression [105]. Consistent with a baseline mitochondria stress response in *dys-1*, the modules uniquely activated in *dys-1* in response to flight include a stress-responsive module (29) [106] that includes the dystrophin-associated protein Dystrobrevin, a neuromuscular function module (18), and a protein translation in muscle module (93) (Figure 6). The activation of these modules suggests that the baseline neuromuscular dysfunction in *dys-1* is exacerbated by the added negative effect(s) of spaceflight on the neuromuscular system. Interestingly, three modules that are inhibited at baseline in *dys-1* do not alter in response to spaceflight (Figure 6B,C). These are module 6, metabolic response to starvation, module 86, response to mitochondrial stress, and module 149, response to iron stress, pH, and starvation [67]. This, again, suggests that the baseline neuromuscular dysfunction in *dys-1* is exacerbated by the added negative effect(s) of spaceflight on the neuromuscular system. This may explain why *dys-1* are significantly weaker in flight than *wt* (small sample size being the other obvious reason).

### 3.5. Genes Predicted to Be Altered in Space Are Predicted to Be Altered by Drugs on Earth

**Figure 7:**
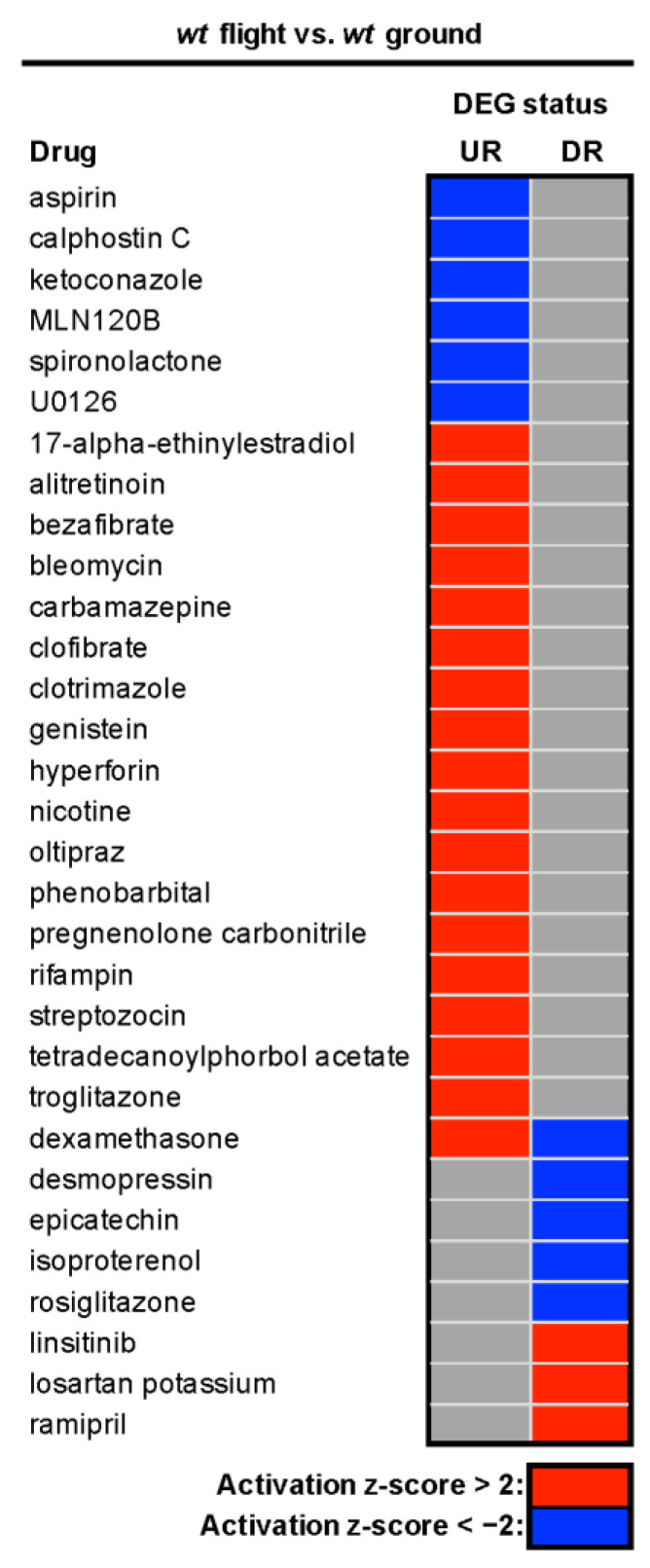
Heatmap of chemical and biologic drug targets predicted to be significantly activated or inhibited for significantly upregulated (UR) and downregulated (DR) genes from the *wt* flight vs. *wt* ground condition, highlighting the potential for alteration in therapeutic potential in the context of spaceflight-induced changes.

With the increased interest in precision medicine in space, the commercialization of space, and the rise of synthetic biology [107,108,109], astropharmacy has recently emerged as a recognized field of specialization [110,111]. The goals of astropharmacy mirror those of pharmacy on Earth but with a specific focus on medication storage [112,113,114,115], access [114,115], use, and manufacturing beyond Earth [111,116]. Current challenges include understanding how detrimental physiological changes in flight might be countered by existing medications, how medications may work differently in flight, and how to ensure our explorers receive the best possible outcome while minimizing side effects despite limited resources. Having found a number of gene expression changes that were conserved with past missions [48,51,52,53,76,77] and profiles indicative of pathways that medications target (e.g., mitochondria, metabolism, neurotransmitters), we were curious if drugs might be predicted to act differently in flight. Therefore, we explored the potential of using drug target predictions to identify compounds that might work better or worse in flight based upon spaceflight-induced gene expression changes. As shown in Figure 7, QIAGEN Ingenuity Pathway Analysis identifies 31 drugs as being regulators of genes whose expression is either increased or decreased in flight. These results suggest, unsurprisingly, that pharmacological activity is likely to be affected, resulting in alterations of medication’s efficacy and safety profile in flight, based upon underlying alterations in gene expression due to not only physiological changes in spaceflight but also further exacerbation by alterations in gene expressions due to medications, such as on the list. Potential consequences include the need for dose adjustments, changes to classic first-line treatment options, and ultimately, for drug selection to be optimized through pharmacogenomics and/or modeling. Clearly, this is an important area of future research as we begin to live and work on other celestial bodies with a more diverse population with different comorbidities needing a better understanding of astropharmacy.

## 4. Discussion

Historically, space biology experiments have been constrained by mass, power, size, and crew time limitations [117]. Now, with a functional ISS, there is functional laboratory equipment, such as the microscope we employed in this study, and crew time for carrying out complex tasks, such as our experiment or extracting and sequencing DNA on ISS [118]. With these improved abilities to conduct science, we were able to extend past studies using *C. elegans* in space to include capture not only of transcriptomic data [48] but to study physiology more comprehensively than simple movement analysis [49]. Using our NemaFlex-S, we were successfully able to measure worm strength both in flight and on the ground. Using these extended capabilities, we were able to test and confirm the hypothesis that worms, like people [119], are weaker in space. This demonstration extends our understanding of muscle response to spaceflight by demonstrating that not only are the molecular changes in muscle sarcomeric gene expression driven by altered MyoD expression conserved between worms and people [51], but so too are the functional consequences. These technical advances and increased knowledge may now be combined to test various nutrient and pharmaceutical interventions to improve muscle strength in flight, as we have recently been able to do for DMD and aging muscle on Earth [46,47]. This experiment presents an example of how near-simultaneous in-flight collection of -omics alongside phenotypic measures can enable insights into the potential molecular mechanisms behind spaceflight-associated physiological deconditioning [120].

Response of gene expression to spaceflight has gained increased interest in the past few years, principally due to the implementation of NASA’s GeneLab program [121]. This has even resulted in introducing network biology into scientific roadmaps, for example ESA’s Biology roadmap 9E [122]. At the simplest level, DEG analysis focuses on individual genes, while this may be useful, it ignores the power of big data, such as reproducibility [123] across experiments and systems and may be biased based upon differences in computational approach/pipeline [124]. For example, the gene identified as most downregulated in this study was previously identified as one of the most upregulated in the first unbiased whole genome response of *C. elegans* to flight [77]. This difference could also be due to operational differences in the two missions. In contrast, our use of GO and network/module analysis has, once again [48], revealed a consistent and reproducible change in gene expression in *C. elegans* in response to spaceflight. A key finding of this study is that strength decreases in flight. As discussed above, declines in mitochondrial gene expression are repeatedly observed in response to spaceflight [48] and are a treatable cause of strength decline on Earth [46,47]. Therefore, testing of mitochondrial interventions to reverse strength decline in space is now required to determine if the correlation between strength and mitochondrial decline is causal or not. Additionally, as our gene annotation and computational skills improve, it is possible to obtain more knowledge from gene expression data than in the past. For example, our module analysis reveals that certain gene expression changes can be attributed to specific tissues (for example, cell adhesion in the hypodermis), whereas others are more universal (for example, mitochondrial stress). Further, our new analysis reveals that calcium metabolism in neurons is also a potential cause of strength decline in space, more specifically by altering acetylcholine signaling, again suggesting an interventional study is required to (dis)prove the casualty of this link. This suggestion, unsurprisingly, has also previously been made for human strength decline in flight [125,126].

As we confront the new age of commercial spaceflight, it remains unclear how experiments such as ours will be possible on as yet unbuilt commercial space stations. The largest space agencies (the Japanese Space Exploration Agency, the European Space Agency, and the National Aeronautics and Space Administration) have not announced any plans for funding scientists to conduct research on such platforms. Given that the US National Lab program relies on scientists to provide their own funding, it seems current US policy may continue, and there will be no funding for academic research on commercial space stations provided by the US government. Clearly, this is an opportunity for smaller agencies, such as the United Kingdom, the Australian, the Italian, and the United Arab Emirates Space Agencies, to grow their research portfolio by continuing to purchase commercial access to these new space stations as they currently do for the ISS. However, for the larger agencies, it is clear that the goal is moving BLEO [36]. For example, NASA’s Thriving in Space initiative [127], the Artemis program [128], and the Commercial Lunar Payload Services (CLPS) program. In this return to the Moon push, the Artemis program is effectively a return to the Apollo program for space biology, which means mass, size, power, and crew time will all be limiting to experiment design again. Similarly, the CLPS program is uncrewed and currently without sample return capabilities. For these reasons, autonomous experiments with data received by telemetry are essential, for example, the recently selected Lunar Explorer Instrument for Space Biology Applications (LEIA) payload. Cube-sats provide an ideal example of how to achieve this, with more than 1500 having previously flown and Biosentinal having already demonstrated that biology experiments beyond low Earth orbit can be successfully conducted [39]. Conducting an experiment such as ours on the Moon or another BLEO destination is technically feasible. Worms have previously been autonomously cultured on ISS for six months, with video data returned via telemetry [70]. Thus, the key challenges to testing worm strength on the Moon are designing culture chambers and strength measurement chambers to function in a cube-sat platform. Currently, funding for the development of hardware for use on the Moon is a challenge via the largest space agencies. Additionally, late load for biology payloads is a key requirement that is currently not accommodated by CLPS, so consideration of inert experiments that can be activated in flight is key. This is possible with *C. elegans* where developmentally arrested larvae have previously been restored to normal development once on orbit [129]. With new destinations for exploration come new challenges for space biology. However, cutting-edge experiments, such as those conducted here with *C. elegans* on ISS, are within technical reality for execution on the Moon or beyond.

## 5. Conclusions

This study extends the growing literature base solidifying the general molecular features underpinning spaceflight-related health decline. By directly linking neuromuscular strength loss with increased stress response and reduced mitochondrial/cytoskeletal gene pathways, we provide a robust framework in which to develop targeted therapeutics against a primary maladaptation to space habitation. Our informatic pipeline, combined with the use of *dys-1* muscle weakness mutants, further highlights perturbed calcium handling and acetylcholine signaling within neurons as primary candidates causing impaired neuromuscular strength in space. These findings provide the foundations for, and an in vivo model of, space-induced strength loss to employ on near-term missions beyond low Earth orbit and to the Moon.

## Figures and Tables

**Table 1:** The effect of space flight on body diameter, length, and muscle strength for adult *wt* and *dys-1* worms.

Strain	Ground				Flight			
	Sample Size	Diameter (µm)	Length (µm)	*f*_95_ (µN)	Sample Size	Diameter (µm)	Length (µm)	*f*_95_ (µN)
*wt*	30	46.97 ± 1.92	1077 ± 60	22.34 ± 5.67	30	44.35 ± 1.88	1062 ± 42	18.62 ± 4.05
*dys-1*	25	48.24 ± 1.87	1110 ± 43	21.38 ± 5.39	29	44.41 ± 2.29	1080 ± 45	14.23 ± 3.87

## Data Availability

Raw RNA sequencing data are deposited in the NCBI Sequence Read Archive with links to BioProject ID PRJNA1026503 (https://www.ncbi.nlm.nih.gov/bioproject/).

## Supplementary Materials

The following supporting information can be downloaded at: https://www.mdpi.com/article/10.3390/cells12202470/s1, Table S1: RNA-Seq_analysis_data.
